# Modelling of soldier fly halteres for gyroscopic oscillations

**DOI:** 10.1242/bio.20149688

**Published:** 2015-01-08

**Authors:** Rizuwana Parween, Rudra Pratap

**Affiliations:** Indian Institute of Science, Department of Mechanical Engineering, Bangalore 560012, India

**Keywords:** Reconstruction of the haltere model, haltere torsional stiffness, haltere bending stiffness, haltere boundary condition, Numerical model of the haltere hinge mechanism

## Abstract

Nature has evolved a beautiful design for small-scale vibratory rate-gyro in the form of dipteran halteres that detect body rotations via Coriolis acceleration. In most Diptera, including soldier fly, *Hermetia illucens*, halteres are a pair of special organs, located in the space between the thorax and the abdomen. The halteres along with their connecting joint with the fly's body constitute a mechanism that is used for muscle-actuated oscillations of the halteres along the actuation direction. These oscillations lead to bending vibrations in the sensing direction (out of the haltere's actuation plane) upon any impressed rotation due to the resulting Coriolis force. This induced vibration is sensed by the sensory organs at the base of the haltere in order to determine the rate of rotation. In this study, we evaluate the boundary conditions and the stiffness of the anesthetized halteres along the actuation and the sensing direction. We take several cross-sectional SEM (scanning electron microscope) images of the soldier fly haltere and construct its three dimensional model to get the mass properties. Based on these measurements, we estimate the natural frequency along both actuation and sensing directions, propose a finite element model of the haltere's joint mechanism, and discuss the significance of the haltere's asymmetric cross-section. The estimated natural frequency along the actuation direction is within the range of the haltere's flapping frequency. However, the natural frequency along the sensing direction is roughly double the haltere's flapping frequency that provides a large bandwidth for sensing the rate of rotation to the soldier flies.

## INTRODUCTION

The remarkable flight characteristics of insects have inspired researchers for more than a century. Researchers have tried to understand the underlying mechanism behind the aerodynamics and kinematics of wings, neural coordination between visual stimulation, neck muscle activation, head rotation of the insects, and flight stability during aerial maneuvers. Insect flight is thus a complex exercise in coordination that is necessarily aided with various sensors. Among insects, dipteran flies detect their body rotations about different axes using both visual and mechanosensory system (Huston and Krapp, 2009; Hengstenberg, 1991). The neural mechanism by which visual organs detect the fly's body rotation is completely different from the mechanism involving mechanosensory organs. Dipteran flies have a pair of special mechanosensory organs, called halteres, that detect the body rotations based on the gyroscopic principle. Halteres are evolved from the hind wings of the four-winged insects like dragonfly. The halteres are inclined backward from the transverse axis of the fly's body at an angle of about 30 degrees that results in non-orthogonality of the two flapping plane.

Halteres were first discovered by Derham (Derham, 1714). Based on his experiments, he found the halteres to be sensory organs that were essential for the flight stability. Later Fraenkel and Pringle also confirmed that a dipteran suffers from flight instability when the halteres are ablated (Fraenkel and Pringle, 1938; Fraenkel, 1939). The function of a haltere was not clear in the scientific community till Pringle's findings (Pringle, 1948). He laid the foundation for the sensory function theory of the haltere system where halteres act as an angular rate sensor based on the gyroscopic principle. He conducted a comprehensive study on the kinematics and dynamics of the haltere, measured the oscillation frequency of the haltere among male and female flies, examined the mass effect on the haltere's flapping frequency, proposed a preliminary model of the haltere joint dynamics, carried out a detailed study of each sensilla group from physiological and mechanics point of view, performed neurological experiments, and measured the influence of the haltere on fight of insects using flash photography. Even though his studies captured essential aspects of the haltere mechanism, it had a few drawbacks. He reported that the halteres were only used to measure the yaw rate component, and did not mention about the importance of the non-orthogonality of the haltere beating planes. Nalbach explained how the non-orthogonality of the haltere beating planes in dipteran insects can measure the rotations about the body's pitch, roll and yaw axes (Nalbach, 1994). He conducted experiments by removing one haltere in the dipteran flies and showed that one haltered animal is inefficient in detecting body rotations. Thus, the halteres were established as vibratory rate-gyros that detect the rate of rotation of the fly's body during aerial rotations (pitch, yaw and roll).

**Fig. 1.**
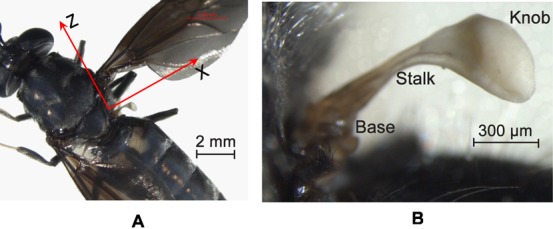
Image of a soldier fly (A), haltere of a soldier fly (B).

Each haltere consists of a long tapered stalk with an end knob and patches of sensory organs, called campaniform sensilla, at the base. Fig. 1 shows the haltere of a soldier fly, *Hermetia illucens*. During flight, the halteres vibrate in the actuation plane with the same frequency as the wings but 180° out of phase. When the fly rotates about any axis during an aerial maneuver, the Coriolis force is induced on the haltere, due to the cross product of the tangential velocity of the haltere and the angular velocity of the body of the fly. This Coriolis force deflects the haltere away from the actuation plane (Nalbach, 1993). These deflections are detected and encoded by the campaniform sensilla, at the haltere base, which act as strain sensors (Fraenkel and Pringle, 1938; Fraenkel, 1939; Pringle, 1948).

During walking and flying, blowflies flap their halteres and do head movements during gaze stabilization whereas no such flapping of the haltere and head movement is observed during standing (Sandeman, 1980). Huston and Krapp also observed that the visual input activates the neck motor neurons but the neck muscles are activated only in the presence of haltere's mechanosensory input (Huston and Krapp, 2009). This implies that there is an interconnection between visual input, haltere oscillation and the head movement. Fox and Daniel claimed that the haltere sensory neurons respond to a stimulation in micro seconds and can activate the neck muscle system which in turn corrects the head position within 3 milliseconds of the sensory input (Fox and Daniel, 2008). During panoramic retinal image stabilizations, the processing of the neuronal signal from the visual system to stabilize the head, takes about 30–100 milliseconds (Huston and Krapp, 2009; Land and Collett, 1974). Thus, the processing of the neuronal signal through the haltere is faster than that of the visual system of the flies. Therefore, Dipteran flies rely on the haltere mechanosensory organ, rather than the visual system, for quicker flight stability. The visual systems are more capable of detecting the slower rotations of the fly (Huston and Krapp, 2009).

The halteres are usually driven with large amplitude cyclic motion at a particular frequency. The actuation frequency is a key component of the Coriolis force induced by the body rotation that is presumably being sensed by the halteres. The energetics of the motion dictates that it is more efficient to use the natural frequency of the haltere for actuation as well as sensing. The halteres are driven at wingbeat frequency, therefore there will be a trade-off between the frequency required for flight and the optimal one for the halteres. Therefore, we would expect the evolutionary processes to evolve the haltere to have natural frequency in the two directions to be either equal to the wingbeat frequency or excitable by the wing beat frequency (as in the case of nonlinear resonance capture). If the insects do indeed use the natural frequency of the haltere, then we are confronted with the questions that how does this simple structure achieve this feat; what enables such large amplitude motion in actuation direction and how does the structure balance the required stiffness in the two directions despite its asymmetric geometry. To answer the question, we reviewed the work done by Chan et al. (Chan et al., 1998). According to their study, the haltere is attached to the metathoracic region by four hard sclerites, which are interconnected by flexible membranous cuticles. These hard sclerites along with cuticular elements form a hinge mechanism that provides large amplitude to the haltere. There are eight direct control muscles and three indirect control muscles attached to the various elements within the hinge. These muscles are used to control the haltere kinematics during various movements. Apart from these muscles, each haltere is connected to a tiny indirect flight muscle (IFM), which runs from dorsal to ventral surface of the fly (Pringle, 1948). This indirect flight muscle acts as an actuator that oscillates the haltere. The exact mechanism by which the input forces from the indirect muscle transmit to the hard sclerites and membranous cuticle and then to the haltere motion is yet to be found. Probably this hinge behaves like a spatial flexural mechanism, in which the four sclerites are rigid bodies and the inter cuticular elements are the flexural joints whose stiffness regulate the haltere motion. This flexural mechanism at the haltere base is able to provide large amplitude to the haltere along the actuation direction.

When the indirect flight muscle is turned on by the neural signal, it starts contracting in an oscillatory fashion, as a result of which the attached haltere also starts oscillating (Josephson et al., 2000). The frequency of the oscillatory contractions does not depend on the frequency of the neural signal, rather it depends on the mechanical properties of the metathorax to which it is attached. Pringle carried out an experiment by inserting a fine platinum wire electrode on the top and bottom region of the thorax and stimulating it by frequency shocks from a neon lamp stimulator (Pringle, 1948). By varying the frequency of stimulation over a range from 40 to 400 shocks per second, he observed no change in the haltere frequency. The haltere frequency was found to be nearly constant and independent of the stimulator frequency. Sellke conducted an experiment by keeping both of the halteres intact and by loading only one of the halteres (Sellke, 1936). He observed that the oscillation frequency of the loaded haltere decreased whereas the oscillation frequency of the unloaded haltere was unaffected. All these experimental observations indicate that the haltere vibrates at its own natural frequency.

Most of the studies done on the haltere system focus on the cellular responses of fly neck motor neurons to haltere (Huston and Krapp, 2009), cellular recordings of haltere motor neurons (Fox et al., 2010), head movement mediation with the haltere (Tracey, 1975), analysis of forces due to various body rotations (Nalbach, 1993), intercellular recordings of the wing and haltere nerves (Fayyazuddin and Dickinson, 1996), mechanism of the activation of the haltere control muscles from visual stimuli (Chan et al., 1998), and head and haltere movement with the imposed angular accelerations (Sandeman, 1980). In this study, we address a completely different aspect of the haltere dynamics that deals with the estimation of the haltere's natural frequency along the driving and the sensing direction. In order to determine the natural frequency of the haltere structure in the two directions, we need to find the boundary conditions, stiffness, and mass properties of the structure in the corresponding directions. We carry out bending experiments and construct an equivalent haltere model with accurate mass distribution in order to get the stiffness and the mass properties respectively. We also discuss the significance of the asymmetric cross-section of the haltere and propose a finite element model of the attachment mechanism at the haltere base.

## MATERIALS AND METHODS

### Materials

We collected two day old, laboratory-reared soldier flies (*Hermitia illucens*). We anesthetized them by cooling in the refrigerator at 4°C for 10 minutes, then prepared the samples as per the requisites of the experiments and the imaging techniques.

To determine the Young's modulus, we removed the halteres from the anesthetized soldier flies. Then, we passed the haltere specimen through a series of increasing alcohol concentrations from 10%, 20%, 40%, 50%, 60%, 75%, 90%, 100%, and 100% alcohol solution about 20 minutes each. We placed the specimen twice in ethanol solution for complete removal of the water content. Thus, we used a small rectangular piece from the stalk of the dehydrated haltere and glued it on a mild steel stub to prepare the sample for nanoindentation tests. The piece selected is representative of the bulk of the haltere material.

For optical microscopic images, we dissected the haltere specimen from the anesthetized soldier fly and kept it on a thin sheet that is ruled with grids with grid spacing of 0.25 mm. We took the images of the haltere by keeping the halteres along two different sides (dorsal and front side).

For scanning electron microscope (SEM) imaging, we dissected the halteres from the anesthetized flies and followed the serial dehydration process. Then, we kept them in desiccators for 72 hours to ensure the complete removal of the water vapour. Then, we coated the desiccated sample with gold film of 10 nm thickness. As we are interested in cross-sectional details of the halteres, we took the images of the haltere at three different sections (the knob, the stalk and the base) using Carl Zeiss AG - ULTRA 55, the Ultra high resolution scanning electron microscope.

To determine the boundary condition and the stiffness of the haltere along the actuation direction, we removed the wings of the anesthetized soldier flies to probe the halteres. However, to determine the boundary condition and the stiffness of the haltere along the sensing direction, we removed the wings and the abdomen of the anesthetized flies.

### Experimental Methods

#### Nano indentation

We determined the Young's modulus of the dehydrated haltere specimens by using a nanoindenter (Triboindenter Hysitron, Minneapolis, USA) with an in situ imaging capability. We have described the detail procedure of the nanoindentaion in our earlier work (Parween et al., 2014). We used a Berkovich diamond indenter with a tip radius of about 100 nm to indent the dehydrated haltere sample. We loaded the haltere specimen up to 160 µN, at a rate of 20 µN/s. We kept the load constant (160 µN) for 0.5 sec and then gradually unloaded the specimen. During the initial stage of unloading, the deformation of the specimen is purely elastic. The slope at the upper portion of the unloading curve is used to determine the Young's modulus (Oliver and Pharr, 2004). We repeated the indentations 10 times at different locations on the sample to obtain the average value of the Young's modulus.

#### Reconstruction of the haltere model

We took the optical images the haltere along the dorsal and front side. In order to get the haltere's cross-sectional dimensions, we took the SEM images at three different sections (the base, the stalk and the knob). By removing all the cuticles at the haltere base, we also took the SEM image of the haltere's attachments to the insect's body. From ImageJ software, we estimate the size of each pixel of the scale bar given in the image and extract the dimensions of the haltere's cross-section at various lengths. In ANSYS Workbench, we construct the cross-sections at various haltere lengths and join these cross-sections through “skin” command. We assign the density and Poission's ratio as 1030 kg/m^3^ (Vincent and Wegst, 2004; Nalbach, 1993) and 0.3 (Wicaksono et al., 2005) to the model respectively and estimate the mass properties (mass and moment of inertia) of the haltere.

#### Micro-Newton static force sensor

**Fig. 2.**
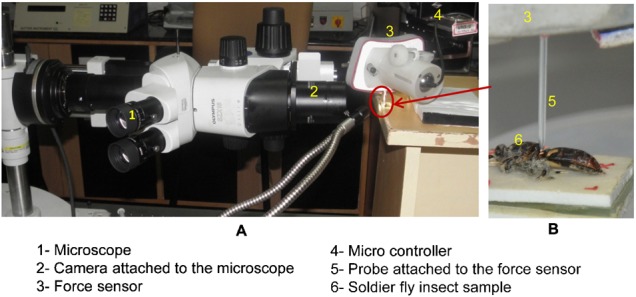
Experimental set up for determining the boundary conditions and the stiffness of the haltere (A), a soldier fly is fixed on the glass substrate (B).

We used a micro-Newton static force sensor set up (Baichapur et al., 2014), as shown in Fig. 2A, to evaluate the haltere stiffness along both directions. This force sensor consists of a compliant mechanism that amplifies the displacement caused by the force to be measured. We used the output displacement, captured with a digital microscope and analysed using image processing techniques to calculate the force with the help of a pre-calibrated force-displacement curve. We mounted the static force sensor experimental set up on a vibration isolation table in order to reduce the effect of unwanted vibration.

#### Calibration of the force sensor

**Fig. 3.**
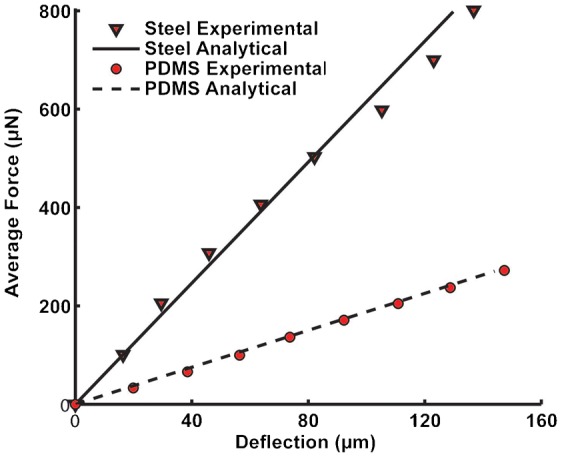
Force-deflection curve for PDMS and spring steel cantilever beams.

To calibrate the force readout from the force sensor, we fabricated two cantilever beams made of spring steel and Polydimethylsiloxane (PDMS), determined their bending stiffnesses using force sensor setup, then compared with their analytical values. We selected these materials as calibration samples because of their well know material properties and consistency of their values. As the haltere is made of soft cuticle, we considered PDMS, a soft material for calibration. To check the repeatability and robustness of the force sensor set up, we also used spring steel for calibration. We fabricated the spring steel cantilever (0.5 mm × 0.5 mm × 80 mm) using wire-cut Electro-Discharge Machining (EDM). We took the Young's modulus of the spring steel as 2.1 GPa from the bulk material specification. We prepared the PDMS samples, by mixing the silicone elastomer base and cross-linker (i.e., hardener) in the ratio of 10:1 by weight. We poured this mixture into plastic moulds and cured at 50°C for a period of 6 hours. We prepared the PDMS cantilever beam of dimension 2.5 mm × 5 mm × 25 mm and measure its Young's modulus as 1.3 MPa using a micro UTM, from MECMESIN. While preparing the samples, we took special care to ensure complete fixity at one end of the beam such that the beam behaves as a cantilever (the slope and deflection at the end is zero). We deflected the tip of the cantilever beam with the help of the probe in the linear range (deflection is less than one tenth of the depth of the beam). We gradually increased the deflection at the cantilever tip and took the corresponding force sensor reading. We repeated the procedure three times for each deflection. Then we plotted the average force against the cantilever tip deflection. From the linear fit of the force deflection curve, we estimated the bending stiffness of the cantilever beam. Fig. 3 shows the force deflection curve for PDMS and spring steel cantilever beams. We also calculated the bending stiffness of the cantilever beam, from the analytical expression:
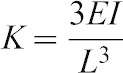
where, *E* is the Young's modulus of the cantilever, *I* is the area moment of inertia and *L* is the length of the beam. Table 1 shows the analytical and experimental bending stiffness of PDMS and spring steel cantilever beams. The experimental bending stiffness of the PDMS and spring steel are close to the corresponding analytical results.

**Table 1.**
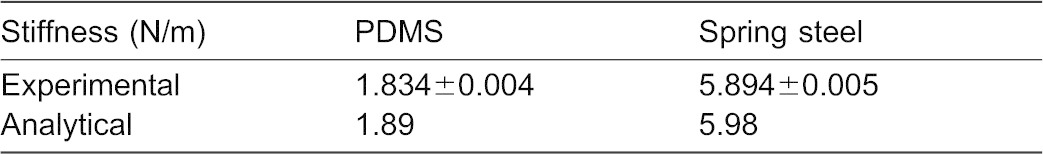
Bending stiffness (N/m) of PDMS and spring steel cantilever beams

#### Estimation of the haltere's stiffness

**Fig. 4.**
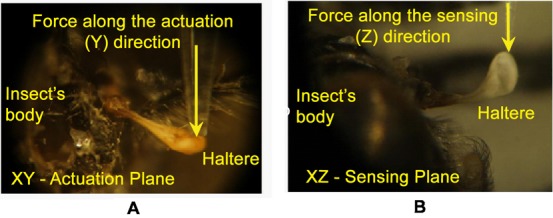
A haltere sample is probed along the actuation direction (A) and along the sensing direction (B).

We used anesthetized wingless flies for all the experiments, conducted for the stiffness measurements. We fixed the anesthetized wingless flies on a glass substrate, with a double-sided tape, shown in Fig. 2B. We place a tiny drop of adhesive under the legs in order to get fixity at the legs and to avoid any movement of the fly's body. While fixing with adhesive, we took enough care to avoid the spreading of the adhesive towards the haltere base. We used a micro pipette of 2 µm diameter, attached to the force sensor, for probing the anesthetized haltere sample along the (Y) actuation direction, as shown in Fig. 4A. Due to the slanting surface of the haltere knob, it was difficult to get a safe probing point on the knob. By a safe point, we mean a point on the haltere knob where we can get sufficient deflection without any slip. We located a safe point by repeated experimentation. In all the experiments, the distance of the probing point was about 1.1±0.15 mm from the base. We ensured that in all measurements, the probe was kept perpendicular to the knob surface to avoid any out-of-plane deflection of the force sensor mechanism. We also observed that at the safe probing point, the anesthetized halteres could be deflected up to 400 µm in the actuation direction. Beyond that, the probe started slipping. The time required to complete an experiment with a single haltere depends on successfully locating a safe probing point on the knob surface. We now describe the experimental procedure used to determine the torsional stiffness of the haltere in the actuation direction.

To measure the torsional stiffness, we displaced the anesthetized haltere from 50 µm to 400 µm with an increment of 50 µm. For example, we displaced the specimen by 50 µm in the actuation direction and recorded the required force. We repeated the procedure three times and estimated the average force. Then, we repeated the experiment with 100 µm displacement. By taking the moment arm length, we calculated the average moment of force and the angular deflection deflection for each increment of the displacement. We plotted the average moment of force with the angular deflection of each haltere sample. From the slope of the linear curve fits of angular deflection versus average moment of force, we estimated torsional stiffness of each haltere. We repeated the above procedure for six different anesthetized haltere samples.

In order to deflect the haltere along the sensing direction, we cut off the wings and the abdomen. We fixed the anesthetized (abdomen less and wingless) flies with a double sided tape and a drop of adhesive on a glass surface. Then, we fixed the glass substrate such that the probe was perpendicular to the haltere knob along the sensing direction (Z) as shown in Fig. 4B. We displaced the haltere from 50 µm to 350 µm with an increment of 50 µm along the sensing direction. For each increment of the displacement, we repeated the procedure three times and measure the average force. Then, we plotted the average force with the linear deflection of the haltere specimen and estimated the haltere's bending stiffness from the slope of the linear curve fits of displacement versus average force. We repeated the above procedure for six different anesthetized haltere samples.

#### Estimation of the haltere's boundary condition

We followed the above fixation procedures in order to determine the boundary condition of the haltere. We deflected the haltere along the actuation (Y) and the sensing direction (Z) by the force sensor and captured the snapshots of the haltere at the corresponding deflected and undeflected positions. Then, we compared the haltere configuration in each case and observed the boundary details.

#### Finite element modeling of the boundary attachment

The stiffness of any structure depends on its geometry, material properties, and the boundary attachment. The torsional and the bending stiffnessess, determined from the static bending experiments, include the contribution of all the three components of the structure. The experiments carried out for stiffness measurement cannot separate the effect of these contributors. But the finite element (FE) model requires them separately. While the geometry and the material properties have been measured separately, we have no way to isolate and figure out the elasticity of the attachment mechanism. This is why we iterate over various boundary attachments and find the correct value of the boundary stiffnessess that lead to the numerically computed values of the torsional and bending stiffnesses matching the experimental results. For FE studies of the haltere's boundary, we discretize the haltere model (3D reconstructed model) with Solid187 element. This element is a ten noded solid 3D with three degrees of freedom at each node: translations in the nodal x, y, and z directions. We consider this element because of its large deflection capabilities and quadratic displacement behaviour, which are well suited for modeling curved surfaces of the haltere.

## RESULTS

### Estimation of the Young's modulus

From nanoindentation of the dehydrated haltere, we obtain the average value of the Young's modulus as 1.5 GPa with a standard deviation of 0.1401 (Parween et al., 2014). These halteres are made of soft cuticle, which is a composite material made of chitin, lipids, and polysaccharides. Depending upon the percentage of the different constituents, cuticles have a range of Young's modulus. Vincent reported that the elastic modulus of the cuticle varies from a few MPa to 2 GPa (Vincent and Wegst, 2004). Klacke and Smith showed that the water content in the cuticles modulates the Young's modulus and the hardness of the three layers (the exo-, meso- and endo cuticle) of the insect cuticle, and also found that there is a ratio of 2.4 between the Young's modulus of the dehydrated and live exocuticle (Klocke and Schmitz, 2011). So, the Young's modulus of the live haltere is 625 MPa, which is used for the finite element analysis.

### Reconstruction of the haltere model

**Fig. 5.**
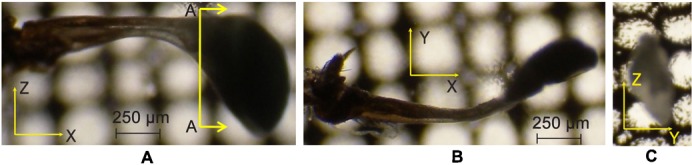
Dorsal view (A), front view (B), and cross-sectional view at AA (C) of a haltere.

**Fig. 6.**
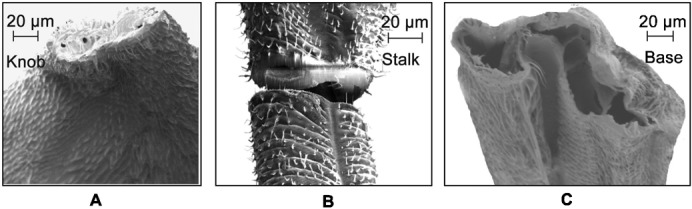
SEM image of the cross-section of the of a haltere at the knob (A), the stalk (B), and the base (C).

**Fig. 7.**
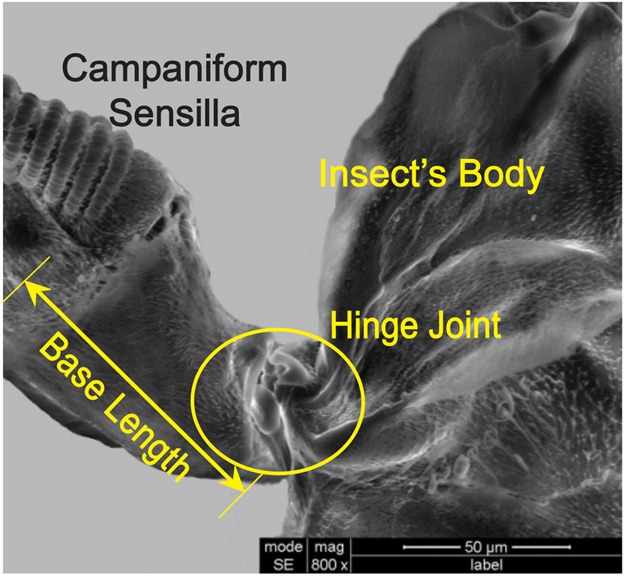
SEM image of the hinge joint at the haltere base.

**Fig. 8.**
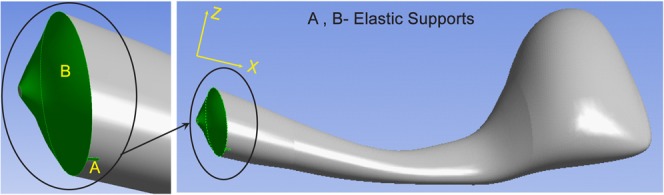
FEM model of the haltere with elastic supports at A and B.

The dorsal view, front view, and the cross-sectional view (at AA) of the haltere are shown in Fig. 5A,B,C respectively. The base is not clearly visible due to the presence of cuticles. From Fig. 5A, the length of the haltere (from the base to the knob) is found to be 1.2 mm. The width of the haltere (along the Z-axis) at the base is 350 µm, which is gradually tapered with a minimum dimension of 130 µm and then gradually increases towards the knob with a maximum dimension of 750 µm. The shape of the haltere knob is more bulky compared to the stalk. From the front view (Fig. 5B), the haltere seems to be a bent structure. The variation in the width in the front side (Fig. 5B) is less compared to the dorsal side (Fig. 5A). The cross-section of the haltere at AA is found to be elliptical, as shown in Fig. 5C. The major and minor diameters of the elliptical cross-section are represented by the width (along the Z-axis) and depth (along the Y-axis) of the haltere respectively. The scanning electron microscopic images of the cross-section of the haltere base, the stalk, and the knob are shown in Fig. 6. The knob is a massive part with a solid cross-section. The haltere stalk is composed of two different tubular structures attached to each other as shown in Fig. 12B. The thickness of the haltere wall and the intermediate joining wall in the stalk is 15 µm and 20 µm respectively. For creating the three-dimensional haltere model, we have not considered the intermediate wall. We consider the stalk to be a hollow structure of wall thickness 15 µm. The cross-section at the base of the haltere is found to be a tubular structure of thickness 10 µm. Fig. 7 shows the SEM image of the haltere's hinged joint. The length of the haltere from the hinge joint to the campaniform sensilla at the base is found to be 60 µm (called the base length). We consider the haltere's cross-section at the hinge joint as elliptical cross-section with minor and major diameter as 10 µm and 27 µm respectively. Based on these dimensions, we construct the 3D model of the haltere (Fig. 8) and obtain its mass and the moment of inertia about the Z-axis as 47.55 µgm and 5.55×10^−14^ kgm^2^ respectively.

### Estimation of the haltere's boundary conditions

**Fig. 9.**
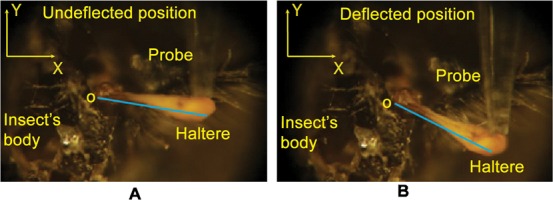
The undeflected position of the haltere, when the probe is touching the knob along the actuation direction (A), the deflected position of the haltere along the actuation direction (B).

In order to get the boundary condition at the haltere base, we deflected the haltere knob of the anesthetized soldier fly along the actuation direction by a probe attached to the micro-Newton static force sensor. Fig. 9 shows the snapshots of the undeflected and deflected positions of the haltere along the actuation direction. We drew tangents at O, located at the haltere base, in the snapshots of the undeflected and deflected positions. We found the angle between the tangent and the adjacent edge of the haltere to be constant at both positions (undeflected and deflected) of the haltere. It is clear that the haltere shows no bending at the base, stalk and the knob along the actuation direction. Thus, the motion along the actuation direction is a rigid body motion.

**Fig. 10.**
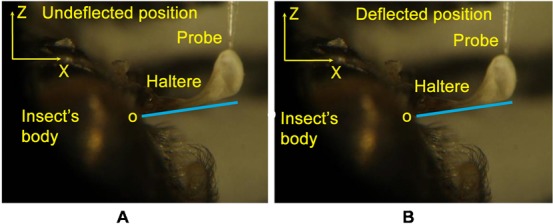
The undeflected position of the haltere, when the probe is touching the knob along the sensing direction (A), the deflected position of the haltere along the sensing direction (B).

**Fig. 11.**
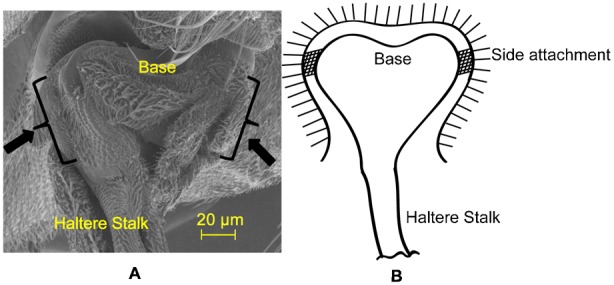
SEM image of the haltere base showing the side attachments.

Similarly, we deflected the haltere knob of the anesthetized soldier fly along the sensing direction. Fig. 10 shows the snapshots of the undeflected and deflected positions of the haltere along the sensing direction. We drew tangents at O, located at the haltere base, in the snapshots of the undeflected and deflected positions. The angle between the tangent and the lower edge of the haltere is less in the deflected position of the haltere as compared to the undeflected position of the haltere. It shows the bending of the stalk and the knob about O in the sensing direction. We now investigate the structure of the haltere base, where it is attached to the insect body, as it has clear effect on the boundary condition of the haltere. The SEM image shown in Fig. 11, shows that the haltere base is attached to the insect body on both sides, as shown by the arrow marks. The length of the attached portion of the haltere base with the insect's body is about 40 µm on each side. These two side attachments act as constraint for any free rotation in the sensing direction causing bending of the stalk in this direction. Thus, the haltere can be modelled as a cantilever beam that bends about these side attachments position along the sensing direction.

**Fig. 12.**
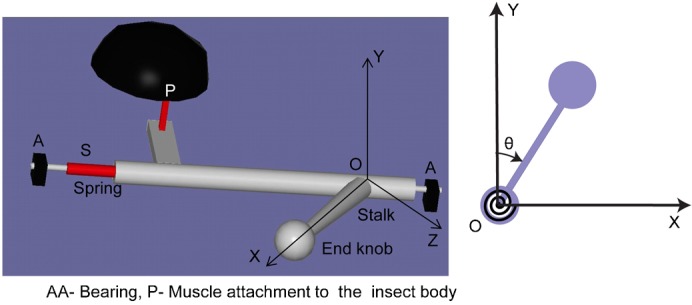
Model of the haltere mechanism.

Based on the above observations, we propose a schematic representation of the haltere mechanism as shown in Fig. 12. The haltere is supported between the bearings AA (Pringle, 1948). The elasticity of the haltere mechanism responsible for the oscillatory motion is represented by a torsional spring (S). The haltere base is connected to the insect body by an indirect flight muscle (Pringle's muscle) attached at point P. When this muscle contracts, a torque is produced, causing the unidirectional rotation of the haltere. During this rotation, the energy is stored in the spring S, which is expended for the reverse motion of the haltere. Thus, the haltere undergoes torsional vibrations in the XY plane as a rigid body and behaves like a cantilever in the XZ plane.

### Estimation of the haltere's stiffness

**Fig. 13.**
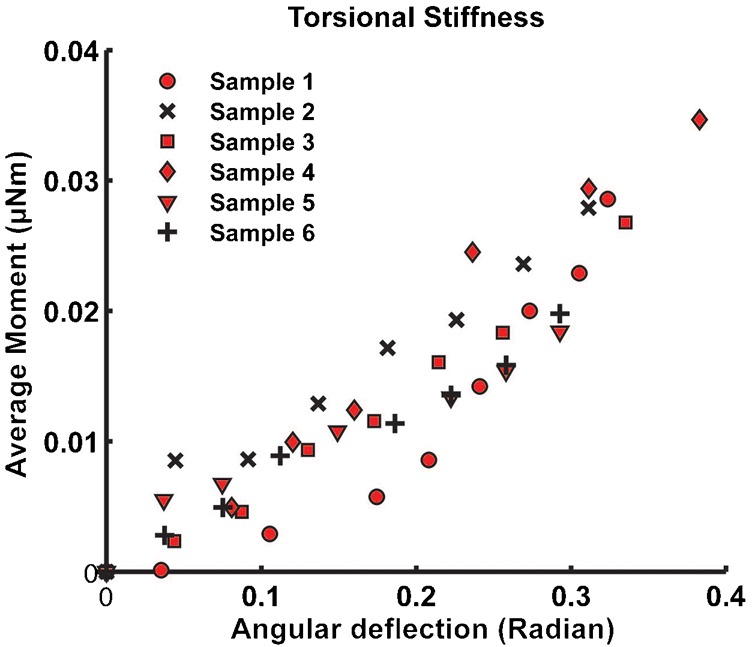
Average moment versus angular deflection data along the actuation direction for anesthetized haltere (the torsional stiffness is determined from the average of fitted stiffness for each data set).

The sample calculations for the torsional stiffness of the anesthetized haltere are shown here. The average force required to deflect the haltere by 50 µm in the actuation direction is 2.12 µN. The arm length and the base length are taken as 1.2 mm and 60 µm respectively (Fig. 7). The corresponding average moment is 0.0027 µNm. The angular deflection is therefore, 0.0376 radian (ratio of the haltere deflection with the moment arm—which is the sum of the arm length and base length). This is how we obtained the data points as shown in Fig. 13. The slope of the linear fit for each set (Fig. 13 contains six data sets) gives the value of the stiffness. The average torsional stiffness is found to be 0.0715±0.01 µNm/radian in the actuation direction for anesthetized flies (Table 2).

**Table 2.**
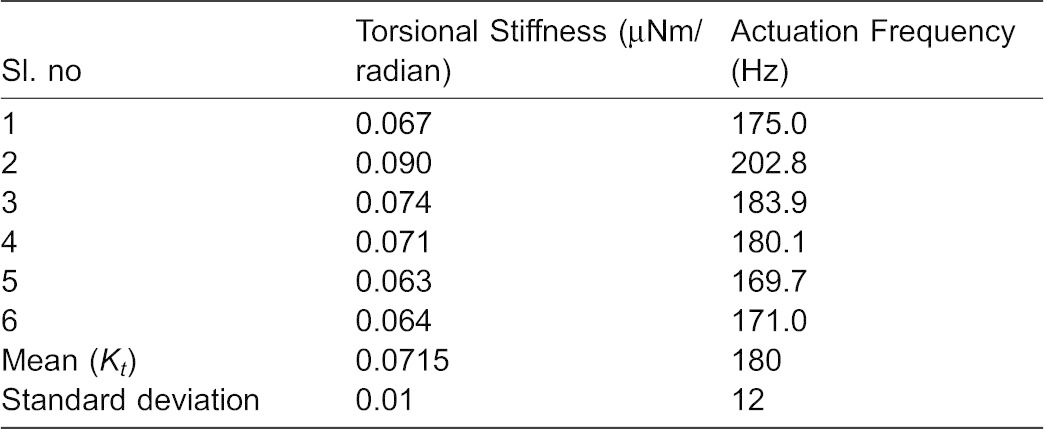
Torsional stiffness (µNm/radian) along the driving direction for anesthetized haltere

**Fig. 14.**
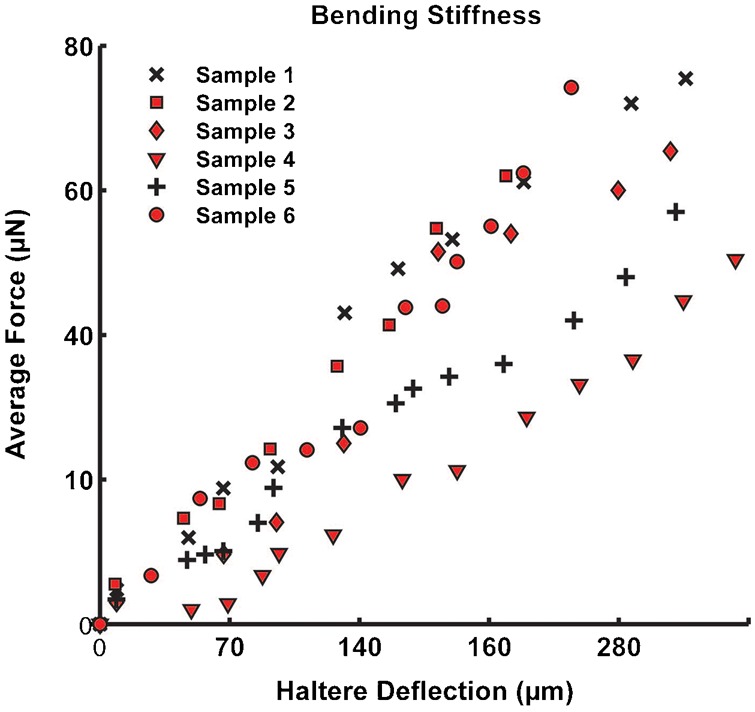
Average force versus haltere deflection data along the sensing direction for anesthetized haltere (the bending stiffness is determined from the average of fitted stiffness for each data).

Similarly, for measuring the bending stiffness, we deflected the anesthetized haltere at the knob along the sensing direction. The average force required to deflect the haltere by 50 µm in the sensing direction is 8 µN. The haltere is found to be very stiff along the sensing direction. Fig. 14 shows the average forces versus deflection data for the anesthetized halteres in the sensing direction. The slope of each force versus deflection curve gives the bending stiffness. The average bending stiffness is found to be 0.223±0.05 N/m in the sensing direction for anesthetized flies (Table 3).

**Table 3.**
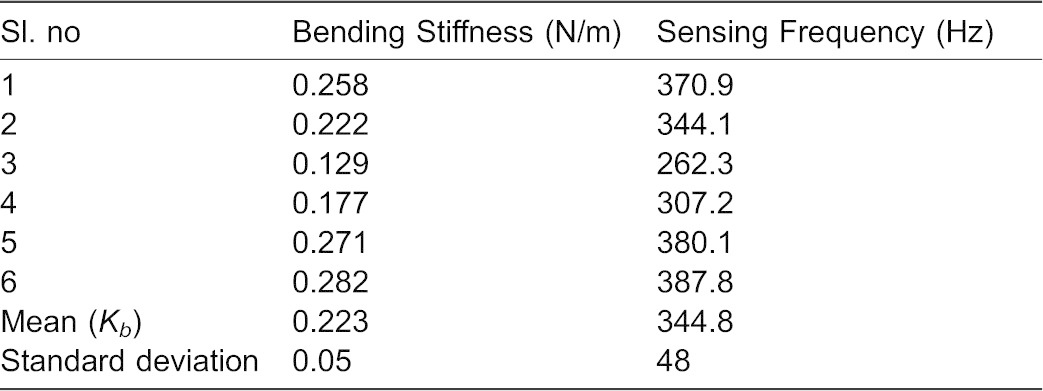
Bending stiffness (N/m) along the sensing direction for anesthetized haltere

**Fig. 15.**
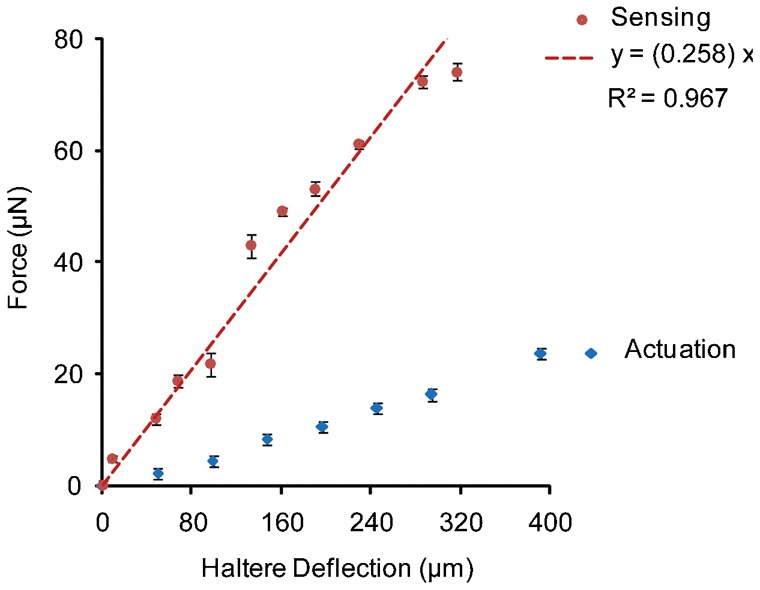
Error plot of force-deflection of one sample along the actuation and the sensing direction.

Fig. 15 shows the haltere's force-deflection data with the error bars along both directions and the slope of the linear fit the force-deflection data along the sensing direction. We considered the initial deflection of the haltere along the actuation direction as 50 µm, for which we obtained the force as 2.12 µN. If we deflect the haltere less than 50 µm, we get a very low force, which is less than the threshold (2 µN) limit of the set up. We also considered the same initial deflection of the haltere (50 µm) along the sensing direction in order to compare the force required with that in the actuation direction (direct comparison). This micro-static force sensor can detect the quasi-static forces from 2 µN to 1400 µN and is calibrated for linearity of the force-deflection data over this range (Baichapur et al., 2014). Even though, the forces measured for the calibration objects are an order of magnitude higher than those for the halteres, we can rely on the accuracy and repeatability of the measurements at low forces due to its linearity behavior in this scope. The scatter in the dataset is because of the variation in the insects used in the experiments and the variance in the measurement as well. The variance in the force data depends on the haltere arm length (distance between the base and the safe probing point on the haltere knob),and the inclination angle of the haltere in the beating plane.

### Estimation of the haltere's natural frequency

Since the haltere is modelled as a rigid body with a torsional spring at the base, it behaves as a torsional pendulum in the actuation direction. Its natural frequency, therefore, can be estimated from:
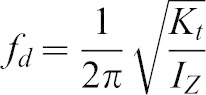
(1)where *K_t_* is the torsional stiffness and *I_Z_* is the mass moment of inertia. The halteres flap in the XY plane (Fig. 12), and hence we need to consider the moment of inertia about the Z-axis. Using the experimentally obtained torsional stiffness of the haltere and the moment of inertia, we obtain the frequency of oscillation as 180±12 Hz. The computed natural frequency of the haltere is within the reported values (105–185 Hz).

Similarly, the natural frequency of the haltere in the sensing direction can be estimated from:
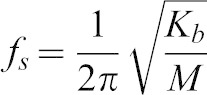
(2)where, *K_b_* is the bending stiffness in the sensing direction and *M* is the effective mass of the haltere. Since the stalk and the base are hollow (Fig. 6), their masses are negligible compared to that of the knob. We approximate the effective mass of the haltere to be the mass of the knob, which is 42.17 µgm. Using the experimentally obtained bending stiffness of the haltere and the mass of the knob, we get the natural frequency along the sensing direction as 345±48 Hz. In Eqns 1, 2, we ignore the possibility of any damping effect. The damping might arise from the internal “friction” in the hinge (for actuation frequency) and the external aerodynamic loading (for sensing frequency). This damping term would likely impact natural frequencies of oscillation along both directions. We assume the damping term to be relatively small and that has minimal effect on natural frequencies along each direction. Thus our calculation shows that the natural frequency along the sensing direction is about twice the natural frequency along the actuation direction.

### Finite element model of the haltere's boundary attachments

Fig. 8 shows the reconstructed model of the haltere with the tapered base (surface B) and the side limiting surface (surface A) as the contact surfaces with the insect's body. We assume these contact surfaces (A and B) to be fixed, which essentially means these surfaces have infinite stiffness (zero translational and rotational the degrees of freedom). From static analysis (in ANSYS workbench), we numerically compute the torsional stiffness along the actuation direction (as per sample calculation) and the bending stiffness along the sensing direction. Then, we compare theses stiffnesses with the experimental results (the torsional stiffness of 0.0715 µNm/radian and bending stiffness of 0.223 N/m). We find this numerical model of the haltere (haltere model with fixed supports at A and B) stiffer than the actual haltere. Therefore, the surface A and B of the haltere's base cannot have infinite stiffness.

**Fig. 16.**
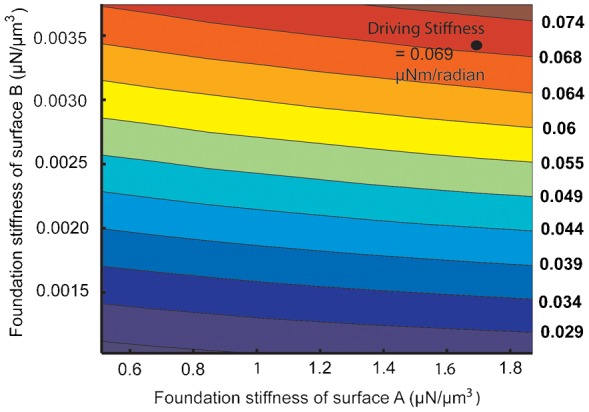
Contour plot of the torsional stiffness with respect to the foundation stiffness of surface A and B.

**Fig. 17.**
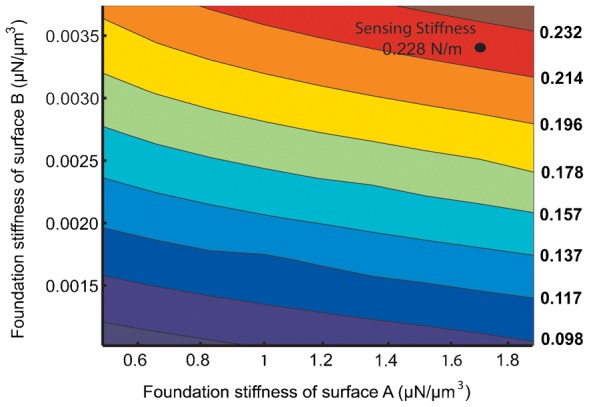
Contour plot of the bending stiffness with respect to the foundation stiffness of surface A and B.

Based on the static bending experiments of the haltere along both directions, there is good evidence that the hinge behaves as a Hookean spring (data represented in Figs 13 and 14). Thus, we consider these contact surfaces to be elastic supports (Hookean contact springs), which deform elastically with respect to the insect's body during haltere motion. It can also be argued from the physiological point of view. The haltere is attached to the metathoracic region by four hard sclerites, which are interconnected by flexible membranous cuticles (Chan et al., 1998). So these cuticles with finite stiffness can provide elasticity to the attachment to the haltere. Such an elastic support on a boundary surface can be easily represented by a linear spring with finite stiffness attached normal to that surface, called foundation stiffness. In this study, we define the foundation stiffness is the pressure required to produce a unit deflection of the elastic surface. In order to propose an accurate numerical model of the haltere, we need to quantify the foundation stiffnesses of the elastic supports (A and B) connecting the haltere to the body. We vary the foundation stiffness of the elastic supports and estimate the stiffness of the haltere in each direction and compare with the experimental data. Fig. 16 shows the contour plot of the haltere's stiffness along the actuation direction with respect to the contact spring at A and B with foundation stiffness K_A_ and K_B_ respectively. Fig. 17 shows the contour plot of the haltere's stiffness along the sensing direction with respect to the foundation stiffness K_A_ and K_B_. From these contour plots, we need to select a pair of values of K_A_ and K_B_ that give us the correct (as determined experimentally) torsional and bending stiffness. As we see from the contour plots, a value of K_A_ = 1.7 µN/µm^3^ and K_B_ = 0.0034 µN/µm^3^ satisfy our requirements. Thus, with foundation stiffnesses, K_A_ = 1.7 µN/µm^3^ and K_B_ = 0.0034 µN/µm^3^, we obtain the torsional stiffness of 0.069 µNm/radian and bending stiffness of 0.228 N/m of the haltere in the actuation and sensing directions respectively. So, the bending stiffness and torsional stiffness of the haltere model are within the range of the experimental results. With the same value of K_A_ and K_B_ , we also obtain the first natural frequency as 180 Hz along the actuation direction, which is close to the analytical value (Eqn 1). Thus, the haltere with elastic support as attachment surfaces at the base seems to be a reasonable model of the haltere joint.

## DISCUSSION

The haltere essentially behaves as a rigid body along the actuation direction. It is found that when the probe is removed, the haltere comes to the original position slowly. From mechanics point of view, when any rigid structure bounces back from a deflected position, the restoring force does not come from the structure, its simply from the elastic elements (cuticles or muscle) present at the base. The elastic elements present at the haltere base behave as energy storing components. Therefore, the haltere's bouncing back along the actuation direction is either due to the elasticity of the cuticles or the muscle attached to the base and it is reasonable to model the underlying actuation mechanism with a rigid stalk connected to a torsional spring at the base.

Along the sensing direction, when the probe is removed, the haltere bounces back to the original position. As it behaves as a flexible body (cantilever) along the sensing direction, the bouncing back occurs due to the elasticity of the haltere structure. Wiesenborn showed that the halteres of the dipteran group contain resilin (Wiesenborn, 2011), an elastic protein (Weis Fogh, 1960). Resilin is a composite material which has low stiffness, high resilience and excellent energy storage capability (Chan et al., 1998). Thus, the elasticity of the haltere structure in the sensing direction is due to the presence of resilin, which restores the bending deformation. Indeed, this pattern would appear to be consistent with the hypothesized function of the campaniform sensilla as strain sensors. That is, for strain sensors to be useful, they need to be placed on a structure that actually deforms under load (as the haltere does due to bending in the sensing direction), rather than a structure that remains rigid (as the haltere is in the actuation direction). Thus, this study shows that the haltere structural behavior corresponds to the hypothesized role of the campaniform sensilla.

Based on the experimentally observed boundary conditions and stiffness measurements, we propose a simple model of the haltere joint with finite stiffness at the contact surface of the haltere, which is attached to the insect's body. The elastic stiffness of the side limiting surface (K_A_) is found to be much greater than the tapered surface (K_B_), commensurate with the fact that the haltere is stiffer along the sensing direction as compared to the actuation direction. However, their values in relation to each other (for example, ratio of K_A_ and K_B_) does not have much meaning because their contribution to the stiffness of the haltere in the two directions is only partial (Stiffness is a property of the geometry, material properties, and the boundary condition of a system).

Sane et al. showed that the antenna of four-winged insects (Hawk moths) is analogous to the haltere of the dipteran flies (Sane et al., 2007). During low-light conditions, moths use a pair of antennae to detect their body rotation, based on the Coriolis principle. From high speed videography, they monitored the motion of the antenna during hovering and found that the flapping frequency of the antenna is same as the wing beat frequency. They also measured the neuronal spiking response of the mechanosensors present at the antennal base. The observations show that the neuronal spiking response has a frequency component that is twice the wing beat frequency (antennal flapping frequency). In case of dipteran haltere, Pringle showed that the Coriolis force has frequency components either at the haltere flapping frequency or twice this frequency (Pringle, 1948). From the experimental observations, we also found the natural frequency of the haltere in the actuation and the sensing direction to be 180±12 Hz and 345±48 Hz respectively. The natural frequency of the haltere along the actuation direction is within the range of the haltere's flapping frequency (105–185 Hz) and consequently within the wing beat frequency. However, the natural frequency along the sensing direction is approximately double the haltere's flapping frequency. In the light of the measured natural frequency in the sensing direction, it is clear that the Coriolis force will exhibit two frequency components – one from the actuation frequency and the other from the sensing frequency.

In the existing MEMS vibratory gyroscopes (Acar and Shkel, 2009), the design of the structure is made in such way that the sense mode natural frequency is slightly away from the drive mode natural frequency. The difference in the natural frequencies along the two directions is called the bandwidth. The bandwidth in a gyroscope is essential for sensing the time varying rotation rates. The larger the bandwidth, the quicker is the response of the device. Even though we get the maximum response along the sensing direction in case of perfect mode matching of the drive and sense mode frequency, such matching leads to zero bandwidth of the sensor. As a consequence, the device takes a long time to respond to the variations in the input rotation rate. Since the flies do various rotations during maneuver, certain amount of mismatch between the actuation and sensing frequency of the haltere is essential for fly's sensing mechanism. In case of haltere, we obtain a large bandwidth (difference in actuation and sensing frequency). Nature has evolved the haltere's cross-section and boundary attachments in asymmetric fashion, which provides a large bandwidth for sensing the rate of rotation to the dipteran flies. Thus, Dipteran halteres represent a smart design of the micro-scale vibratory gyroscope.

### List of symbols

*K_t_*, torsional stiffness of the haltere along the flapping direction; *K_b_*, bending stiffness of the haltere along the sensing direction; *I_Z_*, mass moment of inertia of the haltere; *M*, effective mass of the haltere; *f_d_*, frequency of the haltere along the flapping direction; *f_s_*, frequency of the haltere along the sensing direction; *K_A_*, foundation stiffness of the surface A; *K_B_*, foundation stiffness of the surface B.
